# Structural basis for the broad and potent cross-reactivity of an N501Y-centric antibody against sarbecoviruses

**DOI:** 10.3389/fimmu.2022.1049867

**Published:** 2022-11-17

**Authors:** Bo-Seong Jeong, Joon Young Jeon, Chih-Jen Lai, Hye-Yeoung Yun, Jae U. Jung, Byung-Ha Oh

**Affiliations:** ^1^Department of Biological Sciences, KAIST Institute for the Biocentury, Korea Advanced Institute of Science and Technology, Daejeon, South Korea; ^2^Department of Protein Design, Therazyne, lnc., Daejeon, South Korea; ^3^Cancer Biology Department, Infection Biology Program, and Global Center for Pathogen and Human Health Research, Lerner Research Institute, Cleveland Clinic, Cleveland, OH, United States; ^4^New Target Team, Promedigen, lnc., Daejeon, South Korea

**Keywords:** SARS-CoV-2, viral evolution, key epitope, computational affinity maturation, broadly neutralizing antibody, broad-spectrum vaccine

## Abstract

More than 80% of SARS-CoV-2 variants, including Alpha and Omicron, contain an N501Y mutation in the receptor-binding domain (RBD) of the spike protein. The N501Y change is an adaptive mutation enabling tighter interaction with the human ACE2 receptor. We have developed a broadly neutralizing antibody (nAb), D27LEY, whose binding affinity was intentionally optimized for Y501. This N501Y-centric antibody not only interacts with the Y501-containing RBDs of SARS-CoV-2 variants, including Omicron, with pico- or subnanomolar binding affinity, but also binds tightly to the RBDs with a different amino acid at residue 501. The crystal structure of the Fab fragment of D27LEY bound to the RBD of the Alpha variant reveals that the Y501-containing loop adopts a ribbon-like topology and serves as a small but major epitope in which Y501 is a part of extensive intermolecular interactions. A hydrophobic cleft on the most conserved surface of the RBD core serves as another major binding epitope. These data explain the broad and potent cross-reactivity of this N501Y-centric antibody, and suggest that a vaccine antigenic component composed of the RBD core and a part of receptor-binding motif (RBM) containing tyrosine at residue 501 might elicit broad and potent humoral responses across sarbecoviruses.

## Introduction

COVID-19 is a global pandemic started in December 2019 (1, 2). This disease is caused by SARS-CoV-2, a positive-sense single-stranded RNA virus and a member of the subgenus Sarbecovirus belonging to the genera Betacoronavirus (2, 3). As of August 2022, COVID-19 has caused more than 576 million infections and claimed at least 6.4 million lives worldwide. SARS-CoV-2 has undergone significant antigenic drift, resulting in the emergence and recession of a series of viral variants: Alpha, Beta, Gamma, Delta, Kappa, Epsilon, Eta, Iota, Lambda, Mu (http://www.who.int). Several of these variants exhibit increased infectivity and resistance to neutralizing antibodies (nAbs), elicited by preventive vaccines or natural infection (4–6).

Currently, SARS-CoV-2 Omicron variant (B.1.1.529) or its subvariants (BA.1, BA.2, BA.3, BA.4, BA.5, BA.2.75) are the most prevalent SARS-CoV-2 variant worldwide. In particular, the Omicron BA.4 and BA.5 subvariants exhibit increased neutralization resistance compared with other subvariants (7) and the global spreading trend (8). Omicron, first identified in November 2021 in South Africa, separates it from previously reported variants in that it carries many more mutations than the others, e.g., having 32 versus 16 mutations on the spike protein in comparison with the Delta variant (9). Omicron is highly transmissible, likely due to carrying more than a dozen mutations in the receptor-binding domain (RBD) of the spike protein, which enables it to interact more tightly with the host receptor protein human ACE2 (hACE2) and to evade nAbs elicited in hosts (10). The analysis of hACE2-RBD interaction and structural analysis of the Omicron RBD indicated that Q498R and N501Y mutations on the RBM provide sufficiently strong interaction to enable the generation of many less favorable changes elsewhere (10–13). In particular, the N501Y mutation was reported to enhance the binding affinity against hACE2 and disrupt antibody neutralization (14, 15), consistent with a reverse genetics study showing that the N501Y substitution enhances fitness gains for replication in primary human airway epithelial cells (16). Moreover, the newly emergent variant Deltamicron, a combination of Delta (AY.4) and Omicron BA.1, also contains the N501Y mutation (17). Together, these observations suggest that N501Y is an adaptive mutation and upcoming variants are likely to contain this mutation.

More than 10 broadly nAbs that are effective against many SARS-CoV-2 variants have been developed (12, 18–21), but only a fraction of these nAbs retain the binding affinity and neutralizing activity against Omicron (22). Previously, through a combined computational and experimental approach, we developed D27LEY, a broadly nAb against all concerning SARS-CoV-2 variants, SARS-CoV-1 and pangolin coronavirus (21). We first obtained D27 by computational antibody design using the Rosetta software suite (23), which exhibited low binding affinity for the RBD of SARS-CoV-2 (Wuhan-Hu-1 strain) (21). Subsequently, based on the structure of the Fab fragment of D27 (D27-Fab) in complex with the Wuhan SARS-CoV-2 RBD (D27-Fab–WT RBD), D27LE was developed by extending the complementary determining regions 3 (CDR3) at the tips by 1 or 2 residues and additionally randomizing the flanking residues (21). D27LEY was further developed by computational sequence optimization of four residues near Y501 of the SARS-CoV-2 RBD (21). This N501Y-centric antibody binds to the N501Y-containing RBDs of the Alpha, Beta and Gamma variants with picomolar binding affinity. Despite this affinity optimization for the Y501 residue, D27LEY binds tightly (*K*_D_ < 1 nM) to the RBDs of all other concerning SARS-CoV-2 variants that have a different amino acid at the 501 residue position (21).

Here, we report that the D27LEY antibody binds to the Omicron RBD with subnanomolar affinity. The crystal structure of the Fab fragment of D27LEY (D27LEY-Fab) in complex with the RBD of the Alpha variant (Alpha RBD) provides the structural basis for the broad cross-reactivity of this nAb against other variants including Omicron, which in turn suggests a rational path toward pan-sabecovirus vaccine design.

## Materials and methods

### Pseudovirus neutralization assay

To prepare vesicular stomatitis virus (VSV) pseudotype with the SARS-CoV-2 Spike protein, HEK293T cells plated overnight (3x10^6^ cells per 10 cm dish) were transfected using calcium phosphate with 15 µg plasmid encoding a codon-optimized Spike protein of SARS-CoV-2 wild type, Omicron B.1.1.529 variant or Delta variant with 18-residue deletion at the cytoplasmic tail. At 24 h posttransfection, the cells expressing the Spike protein were infected with rVSV-deltaG-Luc for 1 h. The cells were washed 3 times with DPBS and incubated with 7~10 mL of media (with 10% FBS). At 24 to 48 h postinfection, the media were harvested, filtered with a 0.45 µm filter, and stored at -80°C in 1ml aliquots. For the pseudovirus neutralization assay, serially diluted D27LEY solution was mixed with each of the three types of pseudoviruses. After 1 h incubation, the mixture was added to 293T-hACE2 cells (3x10^4^ cells per well in 96-well plate) which were pre-seeded overnight. The cells were lysed with the PLB buffer (Promega, E1941) after 24 h, and then the luciferase activity was measured by adding LAR II (Promega, E1501). The percent neutralization was normalized to uninfected cells (100% neutralization) and infected cells (0% neutralization), both in the absence of D27LEY. The IC_50_ titers were determined from the nonlinear curves of the log(agonist) versus normalized response using Prism v9 (GraphPad).

### Production and purification of D27LEY

DNA fragments encoding VH or VL of D27LEY were synthesized (IDT) and cloned into a vector derived from the pCEP4 vector (Invitrogen) which contains the CH1-CH2-CH3 sequence of IgG or the CL sequence of the kappa light chain. The resulting vectors, pCEP4(heavy) and pCEP4(k-light), were introduced into CHO-S cells (Gibco) at a density of 6x10^6^ cells/ml using ExpiFectamine (Gibco) to express D27LEY. The cells were grown in the ExpiCHO expression medium (Gibco) for 10 days. The culture supernatant was collected by centrifugation at 12,000g for 1 h at 4 ℃, diluted by half with Protein A binding buffer (PBS, pH 8.0), loaded onto an open column containing Protein A resin (Genscript), and eluted with Protein A elution buffer (0.1 M glycine, pH 3.0). The eluent was then immediately neutralized with Protein A neutralizing buffer (1M Tris-HCl, pH 8.5), and further purified using a HiLoad 26/60 Superdex 200 column (Cytiva) equilibrated with buffer A composed of 150 mM NaCl and 20 mM Tris-HCl (pH 8.0).

### Production and purification of D27LE

A DNA fragment encoding VH of D27LE was prepared by site-directed mutagenesis and cloned into the pCEP4 vector. The expression and purification procedures were virtually identical to those used for the preparation of D27LEY.

### Production and purification of D27LEY-Fab

The VH sequence of D27LEY was amplified by PCR from the pCEP4(heavy) vector and then cloned into a vector derived from the pCEP4 plasmid which contains the CH1 sequence of IgG with an 8x(His) tag. The resulting vector, pCEP4(Fab-8His), was introduced into CHO-S cells together with the pCEP4(k-light), and the transformed cells were cultured similarly as was done for the D27LEY production. The cell supernatant was loaded onto an open column containing Hispur Ni-NTA resin (Thermo Scientific). The column was washed with buffer A containing 20 mM imidazole, and bound D27LEY-Fab was eluted with buffer A containing 200 mM imidazole. The eluted solution was further purified by size-exclusion chromatography using a HiLoad 26/60 Superdex 75 pg column (Cytiva) equilibrated with buffer A.

### Production and purification of SARS-CoV-2 RBD N501Y

A DNA fragment encoding the RBD(N501Y) was prepared by site-directed mutagenesis and cloned into the pCEP4 vector (Invitrogen). The expression and purification procedures were virtually identical to those used for the preparation of D27LEY-Fab.

### Crystallization, structure determination and refinement

Purified RBD(N501Y) and D27LEY-Fab were mixed in a 1.2:1 molar ratio. The complex between the two was purified by size-exclusion chromatography using a Hiload 26/60 Superdex 200 column (Cytiva) equilibrated with buffer A. The complex was concentrated at 11.7 mg/ml, and an initial crystallization screening was performed in 96-well plates by the sitting-drop vapor diffusion method using Mosquito (Spt Labtech). Finally, crystals were grown in a solution containing 200 mM ammonium citrate tribasic (pH 7.0) and 20% (v/v) polyethyleneglycol 3350. For cryoprotection, the crystal was briefly immersed in a mother liquor containing an additional 10% (v/v) ethylene glycol. X-ray diffraction data were collected on the beamline 5C at the Pohang Accelerator Laboratory, Korea, and processed with HKL2000 (24). Model building and structure refinement were carried out using COOT and PHENIX (25, 26).

### Biolayer interferometry

BLI experiments were performed using an Octet R8 system (Sartorius). Biotinylated SARS-CoV-2 Omicron RBDs (ACRObiosystems) or 6x(His)-tagged SARS-CoV-1 RBD (R&D Systems) at 5 nM was loaded onto streptavidin or Ni-NTA biosensor tips (Sartorius), respectively, immersed in Kinetics Buffer (Sartorius) for 120 s. For measuring *K*_D_, the antibodies at five different concentrations were subjected to BLI runs with an association step (for 240 or 360 s) and a dissociation step (for 720 s). The binding kinetics were analyzed with the Octet BLI Analysis 12.2.2.4 software package (Sartorius).

## Results

### D27LEY is a nAb against Omicron

**Figure 1:**
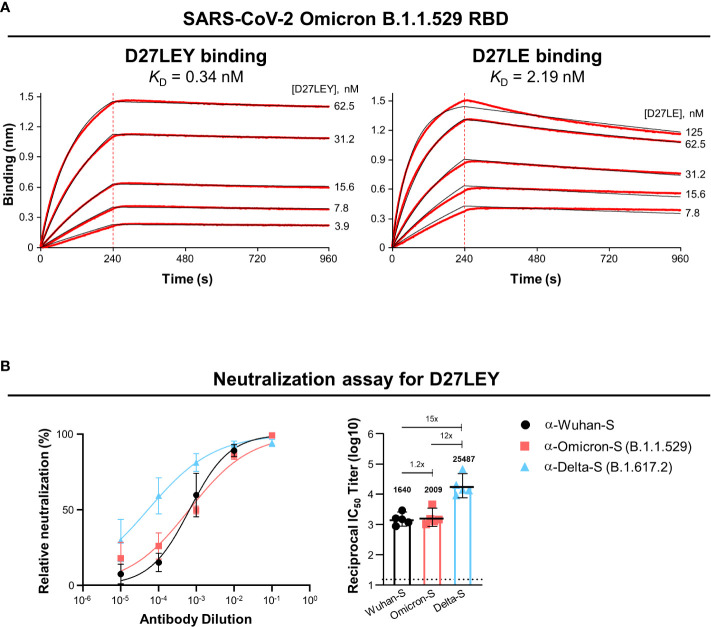
D27LEY binds to the Omicron RBD and neutralizes VSV virus pseudotyped with the Omicron Spike protein. **(A)** Binding affinity. BLI was performed at the five indicated concentrations of D27LEY or D27LE. The experiments were performed in technical triplicate with each giving similar results. Representative sensorgrams are shown. The deduced values of the dissociation constant (*K*_D_) are shown. **(B)** Pseudovirus neutralization. D27LEY (3 mg/mL) was serially diluted and added to the VSV pseudoviruses bearing the Spike protein of the indicated SARS-CoV-2 strains. The mixture was incubated with 293T-hACE2 cells and the luciferase activity was measured. The GMT values with a 95% confidence interval are shown on the right, which correspond to the IC_50_ values of 2.13, 1.96 and 0.18 μg/ml of D27LEY antibody for WT, Omicron and Delta.

Biolayer interferometry (BLI) was used to test whether D27LEY could bind the RBD of Omicron (B.1.1.529). It showed that D27LEY interacts with the Omicron RBD with the *K*_D_ of 0.34 nM, which is a weakened binding affinity compared with that between D27LEY and the Alpha, Beta and Gamma RBDs (*K*_D_ < 0.01 nM) (21), indicating that many mutations on the Omicron RBD negatively affected the binding potency of D27LEY (**Figure 1A**). Consistent with the reduced but tight interaction of D27LEY with the Omicron RBD, vesicular stomatitis virus (VSV) pseudotyped neutralization assay showed that D27LEY conferred strong and consistent neutralizing activities with geometric mean titer (GMT) ranging between 1640 and 2009 against the WT (the Wuhan-Hu-1 strain) Spike protein and Omicron B.1.1.529 Spike protein. D27LEY also exhibited neutralizing activity against pseudotyped virus bearing the Delta (B.1.617.2) Spike protein (GMT of 25,487) (**Figure 1B**). The results suggest that D27LEY is able to broadly neutralize the Spike proteins of all SARS-CoV-2 variants including Omicron.

### Structural characterization of the D27LEY—Alpha RBD interaction

**Figure 2:**
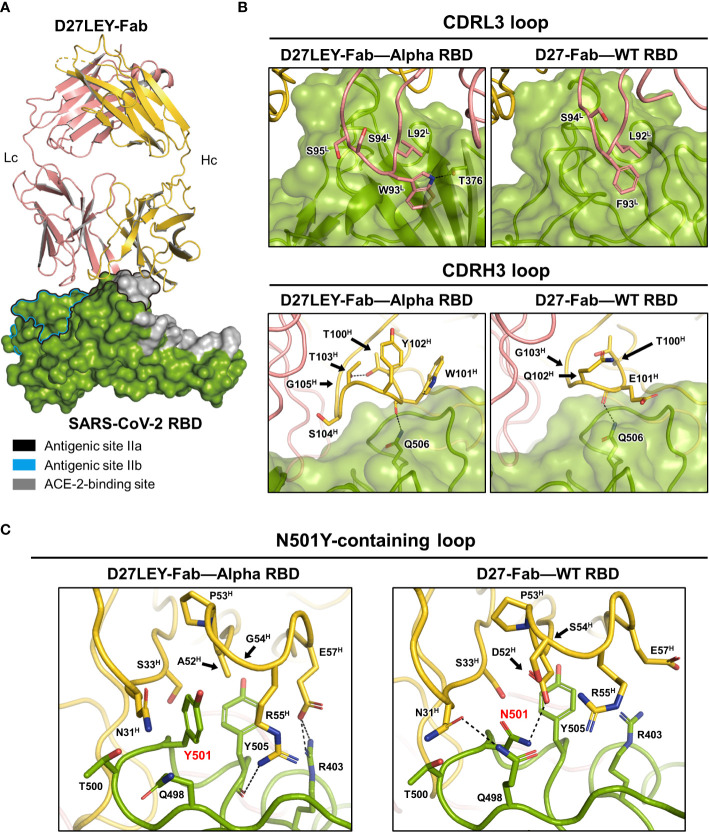
Crystal structure of D27LEY-Fab–Alpha RBD in comparison with D27-Fab–WT RBD. **(A)** Overall view. The RBD-binding interface of D27LEY slightly overlaps with the RBM and largely with the antigenic sites IIa and IIb on the RBD core. **(B)** Side-by-side views of the interactions between the CDR3 loops and the two antibodies (D27LEY and D27). **(C)** The interactions of the N501Y mutation site with D27LEY (Left; Y501) and D27 (Right; N501) are shown side-by-side. The black arrows indicate the two substituted amino acids as a result of the computational affinity maturation (A52^H^ and G54^H^ in D27LEY; D52^H^ and S54^H^ in D27). Note that two unchanged residues, R55^H^ and D57^H^, interact with Y501 and Y505 of the RBD in the D27LEY-Fab–Alpha RBD structure. The RBDs in the D27LEY-Fab–Alpha RBD and D27-Fab–WT RBD (PDB entry: 7VYR) were aligned in **(B, C)**.

To understand the broad cross-reactivity and potent interaction between D27LEY and the RBD variants, we determined the crystal structure of D27LEY-Fab bound to the RBD of the Alpha variant (D27LEY-Fab–Alpha RBD) (**Figure 2A** and **Table 1**). According to the classification of the antibody-binding sites on the RBD (27), D27LEY belongs to the neutralizing antibodies that bind to antigenic site IIa, which includes a conserved surface patch on the RBD core and a small portion of the RBM. D27LEY interacts mostly with this conserved patch and a small portion of the RBM with 6 RBM residues (T500, Y501, G502, V503, Y505, Y508) being the epitope residues. According to another classification, the site IIa-binding antibodies are classified as class 4 antibodies (28, 29).

**Table 1:** X-ray data collection and structure refinement statistics.

Data Collection	D27LEY-Fab‒Alpha RBD
Space group	*P*6_1_22
Unit cell dimensions	
a, b, c (Å)	155.96, 155.96, 166.68
α, β, γ (°)	90.0, 90.0, 120.0
Wavelength (Å)	1.00
Resolution (Å)	38.99-3.51 (3.63-3.51)^a^
R-merge	0.80 (3.27)^a^
I/σ(I)	7.91 (1.38)^a^
Completeness (%)	91.18 (82.25)^a^
Multiplicity	39.6 (41.0)^a^
**Refinement**	
No. of reflections	15,500 (1,519)^a^
Rwork/Rfree (%)	23.7/27.4
R.m.s deviations	
Bond (Å)/Angle (°)	0.002/0.48
Average B-values (Å^2^)	40.03
Ramachandran plot (%)	
Favored/Additional allowed	90.94/8.89
Outliers	0.17

^a^The numbers in parentheses are the statistics from the highest resolution shell.

A comparison of the D27LEY-Fab–Alpha RBD and the D27-Fab–WT RBD structures reveals the structural consequences of the CDR3 loop extensions, which significantly enhance the RBD-binding affinity from 177 nM to 7.12 nM in the KD value (21). D27LEY contains L92^L^-W93^L^-S94^L^-S95^L^ on the CDRL3, while D27 contains L92^L^-F93^L^-S94^L^. Remarkably, the insertion of one serine residue (S94^L^) enables the side-chain of Trp93^L^ of D27LEY to be inserted into a hydrophobic cleft on the RBD core surface encompassing the site IIa, making much more hydrophobic contacts and a hydrogen-bonding interaction in comparison with the corresponding residue Phe93^L^ of D27 (**Figure 2B**). These interactions, which appear as ‘hot spot’ interactions, conceivably contribute to the significantly enhanced binding affinity by the CDRL3 loop extension.

In contrast, the loop extension of the CDRH3 by two-residue insertion does not appear to generate considerably new binding interactions compared with the original CDRH3 of D27. The CDRH3 loop of D27LEY is composed of a six-residue segment (T100^H^-W101^H^-Y102^H^-T103^H^-S104^H^-G105^H^), while that of D27 is composed of a four-residue segment (T100^H^-E101^H^-Q102^H^-G103^H^). The two-residue insertion (Y102^H^, T103^H^) and the E101^H^W and Q102^H^S substitutions of the flanking residues widen the CDRH3 loop, but the resulting loop, which slightly better packs against the RBD, does not show noticeable intermolecular interactions with the RBD core surface (**Figure 2B**). Together, these structural analyses indicate that the interaction between Trp93^L^ and the hydrophobic pocket is a ‘hot-spot’ interaction.

We next examined the structural consequences of the sequence optimization for the N501Y mutation, which resulted in a two-residue change on the CDRH2 loop and drastically enhanced the RBD-binding affinity for the Alpha RBD (having Y501), by greater than 54 folds in the *K*_D_ value (0.54 nM versus at least 0.01 nM) (21). The CDRH2 loop is composed of residues G50^H^-G66^H^, and D27LEY has D52^H^A and S54^H^G substitutions in comparison with D27LE. These two substituted amino acids with the smallest or no side-chain avoid otherwise severe clashes with the bulky side-chain of Y501. Notably, the D52^H^A substitution allows backbone torsional angle changes of E57^H^ such that its side-chain can interact with R403 of the RBD (**Figure 2C**). As a result, A52^H^, E57^H^, R55^H^ (on CDRH2), and N31^H^, S33^H^ (on CDRH1) surround Y501 and Y505 (of the RBD), by making hydrophobic interactions as well as hydrophilic interactions with R403 (**Figure 2C**). Apparently, the observed hydrophobic interactions are extensive in comparison with the corresponding interactions in the D27-Fab–WT RBD structure, where N501 makes hydrophilic interactions and Y505 is mostly exposed (**Figure 2C**). Thus, the extensive intermolecular interactions resulting from the two-residue change provide explanation for the greater than 54-fold increase in the binding potency.

### Modeling D27LEY interaction with the Omicron RBD

**Figure 3:**
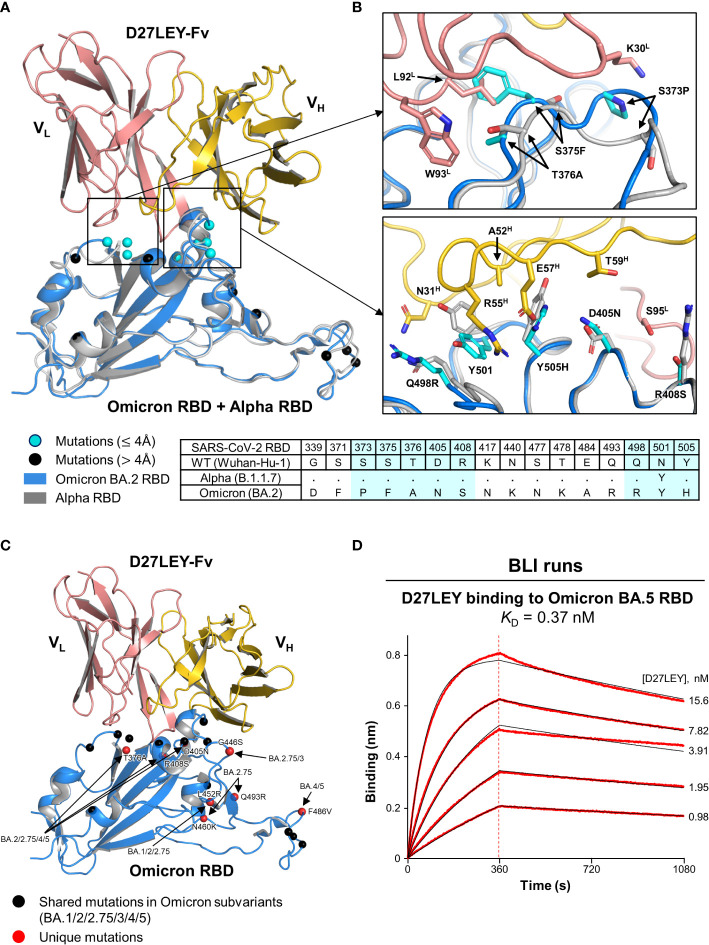
A structural model for D27LEY binding to the Omicron RBD **(A)** Structural superposition of the Omicron RBD (PDB entry: 7ZF8) and D27LEY-Fab–Alpha RBD. The mutations on the RBD of Omicron BA.2 as compared to the RBD of the Wuhan-Hu-1 strain are shown in circles and color-coded according to the interatomic distances from D27LEY. No steric crash is observed between the backbone atoms of D27LEY and the Omicron RBD. **(B)** Enlarged views of the eight mutations that are within 4 Å of D27LEY. The Omicron and Alpha RBDs are shown in grey and cyan, respectively. Side-chain—side-chain clashes between some of these residues and D27LEY are observed, *e.g.*, between H505 (Omicron) and E75^H^ (D27LEY). The amino acid sequences of these two and the wild-type RBDs are compared at the bottom with the eight mutations shaded in cyan. The Alpha RBD differs from the wild-type RBD only at residue 501 in these regions. **(C)** The mutations on the RBD of the indicated Omicron subvariants are shown in circles and color-coded. None of the subvariant-specific mutations is located on the D27LEY-binding surface. **(D)** The binding affinity of D27LEY for Omicron BA.5 was measured by BLI, and the *K*_D_ value is shown.

Initially, we sought to obtain the crystals of D27LEY–Omicron RBD without success. Instead, we built a model of this complex based on the D27LEY–Alpha RBD structure by simple replacement of the Alpha RBD with the structure of the Omicron BA.2 RBD (PDB entry: 7ZF8) (30). The modeled complex showed that 8 out of 16 mutations on the Omicron RBD are close to D27LEY (interatomic distance < 4 Å), and therefore could affect the binding potency of D27LEY: S373P, S375F, T376A, D405N, R408S, Q498R, N501Y and Y505H (**Figures 3A, B**). This number is remarkable, considering the early SARS-CoV-2 variants of concerns (VOCs) whose mutations were entirely or mostly remote from the D27LEY binding interface. These 8 residues are in contact with not the main-chain but the side-chain atoms of D27LEY. Any steric clashes with D27LEY appear to be relieved by adjustment of the torsional angles of the confronting side-chains, explaining the dampened but tight interaction between D27LEY and the Omicron RBD (*K*_D_ of 0.34 nM) compared with the D27LEY-Alpha RBD interaction (*K*_D_ < 0.01 nM). We also modeled the potential interactions between D27LEY and the Omicron subvariants (BA.3, BA.4, BA.5, BA.2.75), showing that the subvariant-specific mutations are not on the D27LEY-binding surface, and therefore D27LEY would exhibit virtually the same binding potency for these Omicron variants (**Figure 3C**). In support of this notion, D27LEY bound to the RBD of the currently dominating subvariant BA.5 with the *K*_D_ of 0.37 nM, which is virtually the same as that for B.1.1.529 (*K*_D_ of 0.34 nM) (**Figure 3D**).

Similar observations were made for a number of broadly nAbs that interact with the RBM at least partially but could resist the RBM mutations on Omicron, including the class 3 antibodies (LY-CoV1404 and BD-744) and the class 4 antibodies (S2X259, Beta-40, Beta-54 and Beta-55) (10, 22, 31–33), which exhibited higher (Beta-40 and Beta-54), similar (LY-CoV1404, BD-744 and Beta-55) or lower than 6 fold decreased neutralization capacity against Omicron in comparison with that against the Wuhan strain or the Beta variant. Together, these observations highlight the plasticity of antigen-antibody interactions and the resulting broad cross-reactivity of class 3 and class 4 antibodies.

### Optimization for Y501 does not reduce the affinity for the RBDs containing a smaller residue at this position

**Figure 4:**
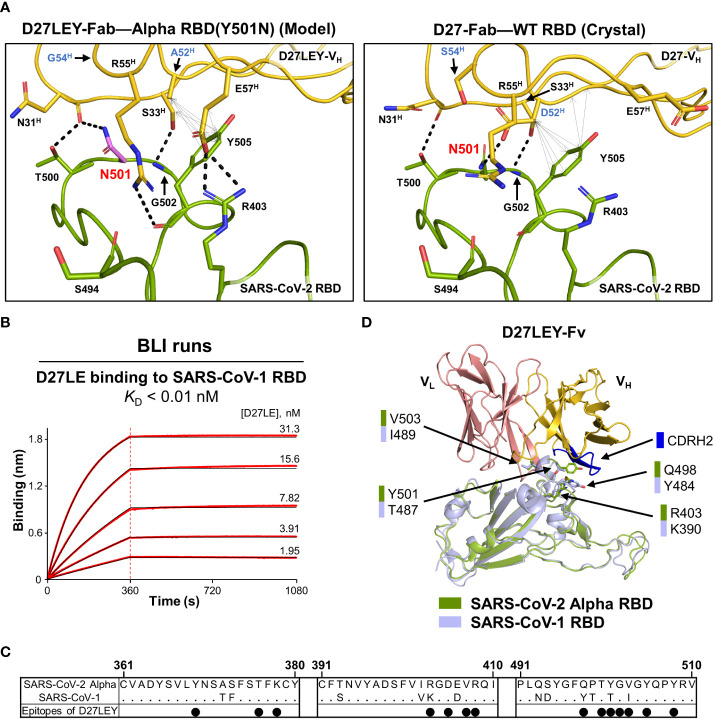
Interaction of D27LEY with RBDs having a smaller amino acid at residue 501. **(A)** N501 (in purple) was modeled on the structure of D27LEY-Fab–Alpha RBD using the Pymol software (Left) and shown together with the structure of D27-Fab–WT RBD (Right), which represents the interaction of D27LE with the WT RBD in this region. The D52^H^A and S54^H^G substitutions in comparison with D27LE are labeled in blue letters. Intermolecular polar and hydrophobic interactions (interatomic distances < 4.3 Å) are indicated by dashed lines and double-headed arrows, respectively, showing more extensive intermolecular interactions engaged by D27LEY than those by D27LE in this region. **(B)** BLI runs for measuring the binding affinity between D27LE and the SARS-CoV-1 RBD. The affinity exceeds the instrumental sensitivity (*K*_D_ < 0.01 nM). **(C)** Sequence alignment of the SARS-CoV-1 and Alpha RBDs. Residue numbering for the SARS-CoV-2 Alpha RBD is shown at the top. The black dots indicate the Alpha RBD epitope residues binding to D27LEY. **(D)** The structures of the SARS-CoV-1 RBD (PDB entry: 2DD8) and D27LEY-Fab–Alpha RBD are aligned. The four epitope residues different in the two RBDs are shown in sticks and labeled. Except T487/Y501, the other three residue pairs are not close to the CDRH2 loop (in blue).

Although the two-residue change (D52^H^A and S54^H^G) in D27LEY compared with D27LE was an optimization for binding to the N501Y RBD mutation, it also resulted in the enhancement of the binding potency for the wild-type RDB from 7.12 nM to 1.14 nM in the *K*_D_ value (21). Simple modeling of N501 in the D27LEY-Fab–Alpha RBD structure shows that the modeled asparagine can be readily accommodated owing to its smaller size. In comparison with N501 in the D27-Fab–WT RBD structure, the modeled N501 residue by itself does not make any better interactions with D27LEY, and thus cannot explain the increased binding affinity (**Figure 4A**). Likely, the favorable interactions of D27LEY with other RBD residues including Y505 and R403, enabled by the two-residue change, would be responsible for the enhanced binding potency of D27LEY for the wild-type RBD. Of note, D27LEY also binds tightly to the PCoV-GD-1 RBD (*K*_D_ of 0.66 nM), which has N501 (21).

SARS-CoV-1 RBD has residue T487, which corresponds to residue 501 in the SARS-CoV-2 RBD. We previously showed that D27LEY binds potently to the SARS-CoV-1 RBD with a *K*_D_ value less than 0.01 nM (21). To test whether the sequence optimization for Y501 also resulted in increasing the binding affinity, we measured the binding affinity of D27LE for the SARS-CoV-1 RBD and found that their interaction was too tight to be measured accurately (*K*_D_ < 0.01 nM) (**Figure 4B**). Sequence comparison shows that four epitope residues, R403, Q498, Y501 and V503, of the SARS-CoV-2 Alpha RBD are substituted with K390, Y484, T487 and I489, respectively, in the SARS-CoV-1 RBD (**Figure 4C**). Mapping these residues on a model for the SARS-CoV-1 RBD bound to D27LEY shows that only T487 is close to the CDRH2 loop, suggesting that the optimized two-residue change (D52^H^A and S54^H^G) on this loop does not affect the D27LEY interaction with other epitope residues of the SARS-CoV-1 RBD (**Figure 4D**). Together, these analyses indicate that D27LEY, generated by the intense N501Y-centric optimization, does not reduce but enhances its local interactions with the RBDs with a smaller amino acid at this residue position.

## Discussion

SARS-CoV-2 has multiple routes of evolution. In addition to the persistent inter-host transmission of the virus, chronic infection in immunocompromised (or in healthy) individuals is an important source of the emergence of new variants by which the virus could acquire mutations and evolve to gain increased infectivity and immune evasion (34–36). Moreover, SARS-CoV-2 is now capable of infecting a variety of animal species, including dogs, house cats and rodents (37, 38), suggesting that these host animals can serve as a large reservoir from which new viral variants can be developed through recombination between different strains similarly as Delta-Omicron recombinant viruses were developed (39). Such variants may spill over to humans, followed by reverse spill over from humans to other animal species in a repeated manner, necessitating advanced preparedness for the future emergence of zoonotic sarbecoviruses, including the development of pan-sarbecovirus vaccines and broadly nAbs.

### Prevalence of the N501Y mutation

The N501Y substitution was noted as a convergent evolution in Brazil, South Africa and elsewhere (40, 41). Consistently, a large-scale study of 506,768 SARS-CoV-2 genome isolates showed that N501Y (or N501T) was a fast-growing RBD mutation (20). According to the CoV-spectrum, as of June 2022, the N501Y mutation had been detected in more than 80% of infections since January (5). Tyrosine residue at this position may remain fixed while the SARS-CoV-2 further evolves, since the N501Y mutation provides fitness gains by enhancing the receptor-binding affinity. As other clades of sarbecoviruses evolve that depend on hACE2 binding for human infection, the corresponding residue position on the spike protein might be also dominated by the tyrosine residue.

### Common features of broadly nAbs which target the RBD

The antigenic site Ia and Ib largely overlap with the RBM, where many different mutations have been found. Therefore, site Ia- and site Ib-binding antibodies (corresponding to class 1 and class 2 antibodies) are rarely cross-reactive across the SARS-CoV-2 variants. In contrast, antigenic sites IIa, IIb and IIc largely overlap with the most conserved surface on one side of the RBD core, and antigenic site IV overlaps with the other conserved surface located on the opposite side (42). Accordingly, site IIa-, site IIb- and site IIc-binding antibodies (corresponding to class 4 antibodies) and site IV-binding antibodies (corresponding to class 3 antibodies) could be cross-reactive across the SARS-CoV-2 variants or other sarbecoviruses. Commonly, nearly all of the known broadly nAbs, including D27LEY, bind to one of the two conserved surfaces exclusively or a small portion of the RBM additionally. The broadly nAbs whose epitope includes the RBM could generate higher antibody responses as they block ACE2 binding to the RBM. Importantly, such broadly nAbs can be elicited by natural infection or vaccination, albeit variably in different individuals.

### Insights into the next-generation vaccine development

The RBD is immunodominant and accounts for 90% of the neutralizing activity of sera isolated from COVID-19 convalescent patients (27). The same study found that antibodies against sites Ia and Ib (class 1 and class 2) were found at high titers in hospitalized donors and in a fraction of non-hospitalized symptomatic and asymptomatic subjects, identifying sites Ia and Ib as the two major antigenic sites on the RBM. The current vaccines (the spike protein either in the form of protein, DNA or mRNA) have intact RBD, and thus would elicit antibodies against site IIa, site IIb, site IIc and site IV (class 3 and class 4) but at lower titers than the two major classes of the elicited antibodies.

To be broadly protective, next-generation SARS-CoV-2 or pan-sarbecovirus vaccines should be able to elicit high titers of broadly nAbs, *i.e.*, those belonging to class 3 or class 4 antibodies. A plausible strategy to develop these vaccines would be to employ, as an antigenic component, a modified RBD in which sites Ia and Ib on the RBM are removed or blocked (*e.g.*, by glycosylation). This modified RBD would promote the elicitation of class 3 and class 4 antibodies while preventing the elicitation of class 1 and class 2 antibodies. It would be rational to keep the residue 501-containing loop intact, as it is a major epitope, and choose tyrosine for residue 501, as it is an adaptive mutation, in the modified RBD.

In conclusion, the presented work identifies that the residue 501-containing loop is a key epitope across the sarvecoviruses, and suggests a rational path for pan-sarbecovirus vaccine development.

## Data availability statement

The data presented in the study are deposited in the Protein Data Bank (PDB) repository, accession number 7YTN.

## Author contributions

B-HO directed the work. B-SJ and JYJ performed the computational design, structure determination and quantification of the binding affinity. C-JL performed the virus neutralization assay, as directed by JUJ. H-YY collected and processed X-ray data. All authors contributed to the article and approved the submitted version.

## Funding

This work was supported by the National Research Foundation grant (NRF-2020R1A4A3079755 to B-HO) through the Korean government (MSIP) and CA200422, CA251275, AI140718, AI140705, AI140705-03S1, AI152190, AI171201, DE023926, DE028521, and Korea Research Institute of Bioscience and Biotechnology Research Initiative Program KGM9942012 to JUJ.

## Acknowledgments

The X-ray data were collected on the Beamline 5C at the Pohang Accelerator Laboratory, Korea. All figures presenting protein structures were generated using PyMOL 2.0.

## Conflict of interest

Authors JYJ and BH-O were employed by Therazyne, lnc. Author HY-Y was employed by Promedigen, lnc.

The remaining authors declare that the research was conducted in the absence of any commercial or financial relationships that could be construed as a potential conflict of interest.

## Publisher’s note

All claims expressed in this article are solely those of the authors and do not necessarily represent those of their affiliated organizations, or those of the publisher, the editors and the reviewers. Any product that may be evaluated in this article, or claim that may be made by its manufacturer, is not guaranteed or endorsed by the publisher.
