# Corrigendum: Structural basis for the broad and potent cross-reactivity of an N501Y-centric antibody against sarbecoviruses

**DOI:** 10.3389/fimmu.2022.1103893

**Published:** 2022-12-08

**Authors:** Bo-Seong Jeong, Joon Young Jeon, Chih-Jen Lai, Hye-Yeoung Yun, Jae U. Jung, Byung-Ha Oh

**Affiliations:** ^1^Department of Biological Sciences, KAIST Institute for the Biocentury, Korea Advanced Institute of Science and Technology, Daejeon, South Korea; ^2^Department of Protein Design, Therazyne, lnc., Daejeon, South Korea; ^3^Cancer Biology Department, Infection Biology Program, and Global Center for Pathogen and Human Health Research, Lerner Research Institute, Cleveland Clinic, Cleveland, OH, United States; ^4^New Target Team, Promedigen, lnc., Daejeon, South Korea

**Keywords:** SARS-CoV-2, viral evolution, key epitope, computational affinity maturation, broadly neutralizing antibody, broad-spectrum vaccine

In the published article, there was an error in the Funding statement. An incorrect grant number was given for the National Research Foundation grant received by author B-HO. The correct Funding statement appears below.

“This work was supported by the National Research Foundation grant (NRF-2020R1A4A3079755 to B-HO) through the Korean government (MSIP) and CA200422, CA251275, AI140718, AI140705, AI140705-03S1, AI152190, AI171201, DE023926, DE028521, and Korea Research Institute of Bioscience and Biotechnology Research Initiative Program KGM9942012 to JUJ.”

The authors apologize for this error and state that this does not change the scientific conclusions of the article in any way. The original article has been updated.

## Publisher’s note

All claims expressed in this article are solely those of the authors and do not necessarily represent those of their affiliated organizations, or those of the publisher, the editors and the reviewers. Any product that may be evaluated in this article, or claim that may be made by its manufacturer, is not guaranteed or endorsed by the publisher.

